# Dosimetric Validation of a GAN-Based Pseudo-CT Generation for MRI-Only Stereotactic Brain Radiotherapy

**DOI:** 10.3390/cancers13051082

**Published:** 2021-03-03

**Authors:** Vincent Bourbonne, Vincent Jaouen, Clément Hognon, Nicolas Boussion, François Lucia, Olivier Pradier, Julien Bert, Dimitris Visvikis, Ulrike Schick

**Affiliations:** 1 Radiation Oncology Department, CHRU Brest, 2 Avenue Foch, 29200 Brest, France; nicolas.boussion@chu-brest.fr (N.B.); francois.lucia@chu-brest.fr (F.L.); olivier.pradier@chu-brest.fr (O.P.); ulrike.schick@chu-brest.fr (U.S.); 2 Laboratoire de Traitement de l’Information Médicale, Unité Mixte de Recherche 1101, Institut National de la Santé et de la Recherche, Université de Bretagne Occidentale, 29200 Brest, France; vjaouen@univ-brest.fr (V.J.); clemhognon@hotmail.fr (C.H.); julien.bert@univ-brest.fr (J.B.); visvikis@univ-brest.fr (D.V.); 3 Institut Mines-Télécom Atlantique, 29200 Brest, France

**Keywords:** brain metastases, stereotactic radiotherapy, GAN, MRI

## Abstract

**Simple Summary:**

Stereotactic radiotherapy (SRT) has become widely accepted as a treatment of choice for patients with a small number of brain metastases that are of an acceptable size. A magnetic resonance imaging (MRI)-only workflow could shorten the planning time and reduce the risk of misalignment in this treatment. Given the absence of a calibrated electronic density in MRI, we successfully compared generative adversarial network (GAN)-generated computed tomography (CT) scans from diagnostic brain MRIs with initial CT scans for the planning of brain stereotactic radiotherapy, finding a high similarity between the planning CT and the synthetic CT for both the organs at risk and the target volumes.

**Abstract:**

Purpose: Stereotactic radiotherapy (SRT) has become widely accepted as a treatment of choice for patients with a small number of brain metastases that are of an acceptable size, allowing for better target dose conformity, resulting in high local control rates and better sparing of organs at risk. An MRI-only workflow could reduce the risk of misalignment between magnetic resonance imaging (MRI) brain studies and computed tomography (CT) scanning for SRT planning, while shortening delays in planning. Given the absence of a calibrated electronic density in MRI, we aimed to assess the equivalence of synthetic CTs generated by a generative adversarial network (GAN) for planning in the brain SRT setting. Methods: All patients with available MRIs and treated with intra-cranial SRT for brain metastases from 2014 to 2018 in our institution were included. After co-registration between the diagnostic MRI and the planning CT, a synthetic CT was generated using a 2D-GAN (2D U-Net). Using the initial treatment plan (Pinnacle v9.10, Philips Healthcare), dosimetric comparison was performed using main dose-volume histogram (DVH) endpoints in respect to ICRU 91 guidelines (Dmax, Dmean, D2%, D50%, D98%) as well as local and global gamma analysis with 1%/1 mm, 2%/1 mm and 2%/2 mm criteria and a 10% threshold to the maximum dose. *t*-test analysis was used for comparison between the two cohorts (initial and synthetic dose maps). Results: 184 patients were included, with 290 treated brain metastases. The mean number of treated lesions per patient was 1 (range 1–6) and the median planning target volume (PTV) was 6.44 cc (range 0.12–45.41). Local and global gamma passing rates (2%/2 mm) were 99.1 CI95% (98.1–99.4) and 99.7 CI95% (99.6–99.7) respectively (CI: confidence interval). DVHs were comparable, with no significant statistical differences regarding ICRU 91′s endpoints. Conclusions: Our study is the first to compare GAN-generated CT scans from diagnostic brain MRIs with initial CT scans for the planning of brain stereotactic radiotherapy. We found high similarity between the planning CT and the synthetic CT for both the organs at risk and the target volumes. Prospective validation is under investigation at our institution.

## 1. Introduction

Approximately 30% of patients with a cancer history [1] and up to 50% of cancer patients, especially those with breast [2] and lung cancers [3], will develop brain metastases. This incidence is increasing due to improvements in cancer therapies and longer overall survival [4]. Stereotactic brain radiotherapy (SRT) has become widely accepted as a treatment of choice in patients with a small number of brain metastases (≤3–5) that are of an acceptable size (≤4 cm) [5]. Indeed, apart from the logistical benefit (3 fractions for SRT versus 10 fractions for whole brain radiotherapy (RT)), SRT allows for better target dose conformity, resulting in both a high level of local control and a better sparing of organs at risk (OAR) [6].

The precision of such a delivery technique requires recent brain magnetic resonance imaging (MRI) for tumor delineation and a computed tomography (CT) scan for planning, performed with a personalized thermoplastic mask. Tumors’ volumes are delineated on the MRI scan and then transferred to the planning CT scan, previously co-registered with the aforementioned MRI scan. Control of the registration is of paramount importance, especially given the low level of metastasis visualization in non-contrast-enhanced CT scans and the risk of misalignment [7].

MRI-based planning could reduce the risk of systematic errors due to misalignments. However, MRI intensities do not possess a calibrated scale; the MRI signal depends on the density of protons and the tissue’s relaxation properties, and not on the electron density like in a CT scan [8,9]. Currently, MRI-only based RT remains cumbersome and is limited to gamma knife therapy, with limited dosimetric planification using the TMR10 algorithm [10].

To take full advantage of the MRI process, an MRI-only workflow could reduce the risk of misalignment between the two imaging modalities and shorten the delays in planning. To this end, synthetic CT scan extraction from diagnostic MRI has recently been developed, allowing for dosimetric planification without the need for a common planning CT scan. This has been studied specifically in prostate cancer and other localizations, including brain tumors [11,12,13,14,15], but clinical and dosimetric data in the specific setting of SRT remain scarce. Given the high dose gradient in SRT, a special consideration with adapted endpoints is needed to assess the feasibility of MRI-only brain SRT.

Several techniques have been developed for the generation of synthetic CT scans from diagnostic MRI. Pseudo-CT can be generated using several approaches (atlas-based or voxel-based) and several calculation techniques (bulk intensity override, neural network (NN), etc.) [13,16,17]. Generative adversarial network (GAN)-generated synthetic CTs have been previously evaluated in the cerebral context but no study has focused on SRT.

Therefore, we aim to assess the equivalence of GAN-generated synthetic CTs for dosimetric planification in the brain SRT setting.

## 2. Material and Methods

### 2.1. Population

We retrospectively included all patients treated with intra-cranial SRT for brain metastases from 2014 to 2018 in our single institution (Radiation Oncology Department, CHRU Brest, Brest, France).

Inclusion criteria were: age ≥18 years old, SRT treatment for one or more brain metastases, brain MRI and planning CT scans realized less than 14 days prior to the treatment delivery. Patients were excluded if the MRI’s field of view (FOV) was judged to be insufficient for tumors and OAR visualization. No limitation was made for the number of lesions treated by SRT.

### 2.2. Imaging

Planning CT (Siemens, Somatom, Siemens Healthcare, Malvern, PA, USA) was obtained with a 1.5-mm slice thickness, a 512 × 512 matrix (pixel size: 0.75 × 0.75 × 1.5 mm^3^), a tube current of 300 mAs, a 120 kV tube voltage and the H30s convolution kernel, with the patient being immobilized with a frameless thermoplastic mask (BrainLAB^®^, Feldkirchen, Germany). No contrast-enhancing agent was used for the planning CT scan.

MRI acquisition was performed on a Siemens 1.5T MRI machine (Siemens Healthcare, Malvern, PA, USA) in supine position, using a 6-channel phased-array surface coil. Anatomical images (axial turbo spin echo T2-weigthed) were combined with functional sequences, such as axial diffusion sequences using several *b*-values and 3D volumetric dynamic contrast-enhanced sequences (T1 sequence with gadolinium injection for Siemens 1.5T). Only the contrast-enhanced T1 sequence was used for the delineation of tumors and certain OARs (brainstem, optic chiasma). Acquisition parameters for the 3D contrast-enhanced T1 sequence were as follows—a repetition time of 450 ms, an echo time of 17 ms, a flip angle of 90°, a slice thickness of 1 mm and a 512 × 512 matrix (pixel size: 0.5 × 0.5 × 1.0 mm^3^) with a 230 mm field of view.

Co-registration between the planning CT and the MRI-T1 was performed using iPLAN RTImage 4.8.1 (BrainLAB^®^, Feldkirchen, Germany) and MRI-T1 images were spatially resampled to the corresponding CT acquisition parameters.

### 2.3. Treatment Planning

Target volume (gross tumor volume, GTV) and OAR were manually delineated by an expert radiation oncologist, with the GTV being defined as the macroscopic contrast-enhanced lesion. The planning target volume (PTV) was based on the GTV, with a 2 mm isotropic margin.

Prescription protocols in our center were previously described [18]. From 2014 to 2016, the prescribed dose (PD) in relation to the PTV was a uniform dose of 3 × 7.7 Gy in the periphery of the PTV and a 99% isodose line covering 99% of the PTV (PD1) with a maximal dose of 107% (24.7 Gy). From 2016 to 2018, the protocol changed to a heterogenous prescription with the creation of a dose gradient inside the PTV—the PD was 3 × 11 Gy at the isocenter, with the 70% isodose line covering 99% of the PTV (PD2). The 99% isodose covered 99% of the PTV in both cases; the only difference was in the dose gradient. The dose calculation grid was set to 2 × 2 × 2 mm.

Dose constraints to OARs remained the same for the two protocols—maximal dose (Dmax) to the optic nerves <13.8 Gy, Dmax to the optic chiasm <10.5 Gy, Dmax to the brainstem <16.8 Gy and V10 Gy (Vx Gy: volume of organ receiving ≥*x* Gy) and V21 Gy to the brain respectively <5% and <20.9 cc. Secondary constraints could also be used if primary goals could not be achieved—for the brainstem, Dmax ≤ 23.1 Gy and V18Gy < 0.5 cm^3^; for optic nerves, Dmax ≤ 17.4 Gy and V15.3 Gy < 0.2 cm^3^. When dose constraints to OARs could not be met, the prescribed dose was decreased to 3 × 7 Gy and 3 × 10 Gy, respectively.

Treatment was delivered using volumetric modulated arc-therapy (VMAT) on a linear accelerator (Truebeam^®^ STx Novalis) equipped with a Millennium MultiLeaf Collimator with 120 leaves (thickness of 2.5 mm at isocenter and up to 8 cm, followed by a thickness of 5 mm from 8 to 22 cm). Every patient was planned using a flattening-filter (FF) VMAT technique with 6-MV beams in the Pinnacle^®^ v9.10 treatment planning system (TPS) (Philips Healthcare, Eindhoven, The Netherlands). Two arcs from 182° to 178° were used, the maximum dose rate being set to 600 MU/min (MU = monitor unit).

### 2.4. Pseudo-CT Generation

A deep conditional generative adversarial network (cGAN) based on the pix2pix architecture was employed to generate synthetic CT scans from MRI scans [19]. A cGAN is composed of a generator network producing fake CT images trained in competition with a discriminator network distinguishing real from fake CT images. The generator was based on the convolutional U-Net architecture [20] and guided by a cost function composed of a pixel loss, penalizing pixel-wise differences with the real CT scan, and of an adversarial loss, encouraging the generator in fooling the discriminator, while penalizing the discriminator accordingly. The original pix2pix implementation was adapted using Keras to treat floating point medical image data instead of 8-bit color images. A two-dimensional approach was considered by feeding axial slices to the network, given the voxel anisotropy of both CT and MRI scans [21]. Before being fed to the network, the co-registered slices were resliced into 256 × 256 voxels using linear interpolation, allowing for a 7-layer U-Net generator architecture with skip-connections. A 16 × 16 PatchGAN architecture was chosen for the discriminator. To suppress the effect of outliers, CT scans were clipped within the interval [−500; 1000] Hounsfield unit (HU) and MRI voxel intensities were clipped to be inside the [1%; 99%] quantile range. A linear stretch of the voxel intensities between values [−1; 1] was then performed to stabilize network training. The parameters of the transformations were applied in reverse order after passing through the network to restore the original image range. The network was trained for 300 epochs using 3510 paired MRI-CT axial slices from 20 patients, defining the training cohort, with a batch size of one, using a Titan graphical processing unit (NVIDIA, Sunnyvale, CA, USA). The remaining patients were considered as the testing cohort.

### 2.5. Dose Calculations

Each synthetic CT scan was affiliated to its corresponding initial CT scan, using the initial iPlan^®^ co-registration. The new dose map was calculated using the replanning tool called Dynamic Planning^®^, implemented in Pinnacle^®^ TPS and the initial dose computation algorithm (Adaptive Convolution). This tool allowed us to recalculate the dose using the same initial radiation plan (with the corresponding isocenter, beam configuration, MU, etc.) without reoptimization. However, as the synthetic CT scan was generated from the already CT-aligned MRI scan, the automatic registration by Dynamic Planning^®^ was canceled to prevent dose miscalculations due to new misalignment errors.

Thus, for each patient, one initial CT scan with the corresponding dose map (initial) and one synthetic CT scan with the calculated dose map (synthetic) were available.

For comparison purposes, the synthetic dose map was then transferred to the initial CT scan using the “dose transfer” tool in MIM Maestro^®^ v7.0.0 (MIM^®^ software Inc., Cleveland, OH, USA).

### 2.6. HU-Comparability

For the comparison between CT-scans, two classes were pre-defined—a “Bone” class including all pixels with an HU-value superior to 150 (HU: Hounsfield unit) and a “Soft-Tissue” class encompassing all pixels with an HU value between −150 and +150. For these 2 classes, both the initial and the synthetic scans were compared using the root mean square error (RMSE) [22], defined as the square of the pixel-wise difference between the predicted (I_pred_) and true (I_true_) image over a region of interest containing n pixels:

$$RMSE=\;\sqrt{\frac{\sum_{N}{\left(I_{pred}-I_{\mathrm{true}}\right)}^{2}}{N}}$$

### 2.7. Digital Reconstructed Radiograph (DRR) Comparability

Digital reconstructed radiographs were created in MIM Maestro^®^ for 20 randomly selected patients and compared using the root mean square error (RMSE) based on the “bone” class.

### 2.8. Gamma Analysis

Three-dimensional gamma analysis was performed using the “SciMoCa™ dose comparison tool” natively available in MIM Maestro^®^. Developed in partnership with Scientific RT^®^, MIM SureCalc^®^ Monte Carlo and its inherent tool (i.e SciMoCa™ v1.0, Scientific RT) provide validated quality assurance [23,24]. Three-dimensional volumetric calculation was performed for both local and global gamma analysis.

As routine parameters, we used 2 mm and 2% with a dose threshold of 10% to the maximum dose as our cut-offs. The gamma passing rate was set as 95% to be successful, for both the local and global gamma analyses.

We also computed local and global gamma analyses with other criteria, 2%/1 mm and 1%/1 mm, with a 10% threshold to the maximum dose.

### 2.9. Dose-Volume Histogram

Dose-volume histograms (DVHs) were extracted from each initial and synthetic dose map for the PTVs and the OARs, following the ICRU 91 dose report [25].

Maximum dose (Dmax) and mean dose (Dmean) were collected for OARs (optic chiasma, optic nerves, eyes, lenses, inner ears and hypophysis). For th brainstem, volume, Dmax, Dmean and V18 Gy were collected. For the brain, volume, minimum dose (Dmin), Dmax, Dmean, V10 Gy and V21 Gy were reported (Vx Gy: volume of the target above the threshold dose x Gy). D2% (Dx% = dose received by x% of the volume), D50% and D98% were collected for all OARs.

Regarding the PTVs, volume, Dmin, Dmax, Dmean, D2% (PTV_D2_), D50% (PTV_D50_) and D98% (PTV_D98_) were measured.

The homogeneity index has several definitions—homogeneity index 1 (HI1 = PTV_Dmax_/PD), homogeneity index 2 (HI2 = (PTV_D2_ − PTV_D98_)/PD) and homogeneity index 3 (HI3 = (PTV_D2_ − PTV_D98_)/PTV_D50_) [26,27,28].

The conformity index is defined by the volume of the reference isodose (reference dose = PD) multiplied by the volume of the prescription isodose, divided by the PTV volume within the prescription volume [25].

The gradient index is defined by the prescription isodose volume at half the prescription isodose, divided by the full prescription isodose volume.

The prescription isodose volume and number of treated brain metastases were also collected. The reference isodose volume was defined as the 23.1 Gy isodose volume. For patients treated with lower doses (3 × 7 Gy or 3 × 10 Gy), we used the 21 Gy isodose volume as the reference isodose volume.

### 2.10. Statistical Analysis

Our objective was to study the equivalence of the synthetic CT scan when compared to the initial dosimetric CT scan for planning, based on the local gamma analysis passing rate. Thus, for a power of 95%, a two-sided alpha risk of 5%, expected standard deviation to the outcome of 5 and an equivalence limit of 3146 patients were needed [29].

Comparison between gamma analysis rates and DVH values was performed using a two-sided *t*-test [30]. Correlation between gamma analysis passing rates and clinical and dosimetric features was tested using Spearman’s coefficient. Such analyses were performed on the overall cohort and then separately on the training and testing cohorts. Statistical analyses were performed with MedCalc^®^ v14.8.5.

## 3. Results

### 3.1. Population

From the 210 patients treated at our institution between 2014 to 2018, 184 patients were included. Twenty-six patients were excluded due to unavailable MRI imaging. With a median age of 60 years (31–85), 42.4% were treated with PD1 and 57.6% with PD2. The three main primary histologies were lung (61.4%) and breast (16.3%) cancers, as well as melanoma (11.4%). Other histologies were kidney (2.7%), gastro-intestinal (6.5%), bladder (1.1%) and osteosarcoma (0.5%) tumors.

With a mean number of treated lesions by patient of 1 (range 1–6), 290 brain metastases were treated, with a mean PTV volume of 6.44 cm^3^ (0.12–45.41).

The main patients’ characteristics are summarized in Table 1.

### 3.2. HU Comparability

The comparability between the two CT-scans based on the HU values was high, with mean RMSE values of 175.50 HU +/− 63.15 and 13.54 +/− 1.96 for the bone and soft-tissue classes, respectively (Table 2).

### 3.3. DRR Comparability

The comparability between the two CT DRRs based on the HU values was high, with a mean RMSE value of 86.16 HU +/− 19.80 and a median RMSE of 80.30 HU. An example is available as Supplementary Figure S1.

### 3.4. Local and Global Gamma Analysis

With the 2%/2 mm cut-off, the mean local gamma analysis passing rate on the overall cohort was 99.1% IC95% (98.1–99.4), with no statistical difference between the training cohort (99.3%) and the testing cohort (99.1%), *p* = 0.43. Results are presented in Table 3.

**Figure 1:**
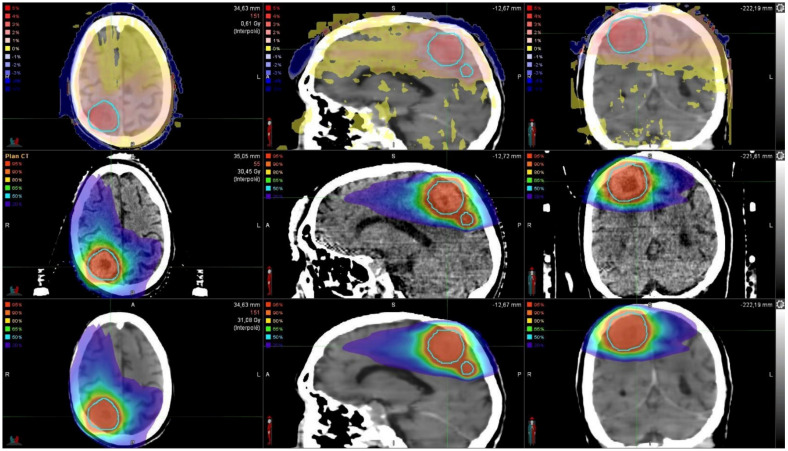
Dose comparison between the initial dose map (**middle**) and the synthetic dose map (**bottom**). Gamma analysis between both dose maps (**top**). PTVs (*n* = 2) are highlighted in light blue.

A comparison of the initial and the synthetic dose maps and the corresponding 3D gamma analysis comparison for a patient can be found in Figure 1.

The mean global gamma analysis passing rate for the overall cohort was 99.7% CI 95% (99.6–99.7), with no statistical difference between the training cohort (99.8%) and the testing cohort (99.7%), *p* = 0.83 (Table 3).

As expected, the local gamma analysis passing rate significantly decreased with more stringent criteria from 99.5 to 98.0 and 87.1 for the 2%/2 mm, 2%/1 mm and 1%/1 mm criteria, respectively. No significant differences were observed for the global gamma analysis passing rates when comparing the three criteria (Table S1).

### 3.5. DVH Comparisons

Regarding OARs, no significant difference was observed for all studied OARs (Table S2). For optic nerves, eyes, lenses, inner ears and hypophysis, mean absolute differences between the initial dose and the synthetic dose were very low, ranging from 0 to 0.19 Gy for Dmax and from 0 to 0.03 Gy for Dmean.

Regarding the brainstem, no significant difference was observed between the initial and synthetic dose maps for Dmax, Dmean and V18 Gy (*p* = 0.93, 0.94 and 0.96 respectively). Seemingly, DVH parameters corresponding to the brain were similar for the two modalities. The highest absolute difference was observed for Dmax, with a mean of 0.42 Gy and a median of 0.44 Gy, representing 1.3% and 1.4% of the mean Dmax to the brain. The local gamma analysis passing rate was not significantly associated to the Dmax to the brain (nor the PTV), with a Spearman’s correlation coefficient of 0.07, *p* = 0.25.

Concerning DVH parameters for the PTV, no significant differences were observed, regardless of the studied feature (Dmax, Dmean, Dmin, D2, D50, D98, etc.), as presented in Table 4.

No significant differences regarding the mean absolute difference for the reference isodose volume nor the studied indexes (conformation index, homogeneity indexes and gradient index) were found between the two dose maps. The mean difference for the reference isodose volume was as low as 0.6 cm^3^ for a mean volume for the reference isodose of 14.43 cm^3^.

**Figure 2:**
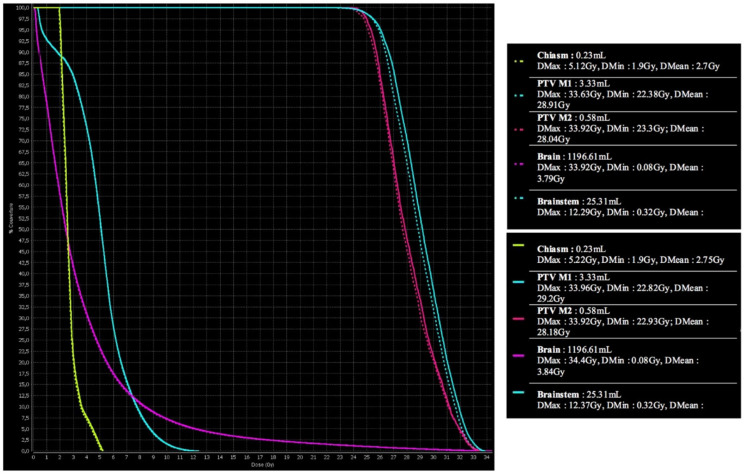
DVH comparison between the initial dose map (top) and the synthetic dose map (bottom). Abbreviations: PTV: planning target volume, M1: metastasis 1, M2: metastasis 2, Dmax: maximum dose, DMin: minimum dose, Dmean: mean dose.

A DVH comparison between the initial and the synthetic CT scans is presented in Figure 2.

## 4. Discussion

To our knowledge, our study is the first dosimetric study of MRI-generated CT scans using GANs in the context of brain metastases treated by SRT. Studies regarding pseudo-CT use, despite being numerous, remain limited to small cohorts. With 184 patients, this is also one of the largest cohorts studying MRI-only RT.

As previously stated, several techniques have been developed for the generation of synthetic CT scans for diagnostic MRI, including GANs, but no studies have focused on SRT [13,16,17]. Based on a 77-patient population, an NN-based algorithm (2D U-Net) was able to produce synthetic brain CT scan imaging with promising results regarding both the Hounsfield unit intensities and the dosimetric evaluation (OARs only) in a study conducted by Kazemifar et al. [12]. Using the same network, we achieved similar results with non-significant differences between the initial and the synthetic scans, with comparable mean absolute differences for all OARs. We also achieved non-significant differences (D2%, D98%, Dmean, Dmax) for all target volumes, irrespective of PTV volume. No synthetic CT scan was rejected based on the global or the local gamma analysis passing rate of 95%. Kazemifar et al. assessed the dosimetric feasibility of MRI-only brain RT, but they only focused on volumetric modulated arc therapy with normofractionated regimens. Moreover, they only reported the D5% and D95%, which are less representative of dose distribution in SRT treatments according to the ICRU 91 report. Beyond DVH parameters, dose distribution can also be assessed via several indices, such as conformation index, homogeneity index and gradient index. In our study, no statistical difference was found regarding each index.

Although gamma analysis is the most used endpoint for dosimetric comparison, it suffers from great heterogeneity in the selected criteria and in accepted passing rates [31].

With the high conformation and dose gradient in SRT, especially with non-homogeneous dose delivery techniques, doses to the OARs are often low, resulting in tight absolute differences and high global analysis passing rates being expected. When focusing on target volumes, global gamma analysis might be insufficient for several reasons.

The cut-off values vary greatly from one center to another. Based on 210 patients treated by VMAT for prostate cancer, testing two different dosimeters and five different criterion duets (3%/3 mm, 2%/2 mm, 2%/1 mm, 1%/2 mm and 1%/1 mm), correlations between the global and local gamma passing rates varied greatly according to the dosimeter type, linac type and gamma criteria [32,33]. It must be noted that the threshold value for high dose was unknown. Acknowledging this heterogeneity, we tested several criteria to support the model comparison.

Global gamma analysis does not take the steep gradient dose into account and is insufficient to correctly evaluate SRT treatment with the commonly used criterion (3%/3 mm) [34].

Although the 2% /2 mm criterion remains widely used in clinical practice [35,36], no clear international guidelines exist. In the special setting of stereotactic ablative RT delivered by VMAT, a 2D global gamma analysis with a 2%/1 mm criterion provided promising results [37]. Comparison between such criteria and our workflow seemed to be of particular interest. Thus, we tested the 2%/1 mm criterion, finding a high comparability between the synthetic dose map and the reference dose map.

Using criteria presented by Andreasen et al. [38], namely 1%/1 mm and 10% dose threshold, and a 3D gamma analysis, the local gamma analysis passing rate with a 1%/1 mm criteria was significantly lower (when compared to 2%/2 mm or 2%/1 mm—87.4 vs. 99.1 vs. 97.99, respectively). The global gamma analysis passing rate with the same criteria remained high, resulting in clinically acceptable differences between the two dose maps in our study (mean global gamma analysis of 99.2 % with the 1%/1 mm criteria). However, the clinical significance of such criteria in SRT treatment remains unknown.

Our workflow relied on a single co-registration performed on iPLAN^®^, used for the treatment planning. The same registration was then used for the synthetic and initial scans. Such a workflow efficiently reduces the risk induced by repeated registrations. Registration is often described as a critical step in multimodal treatment planning [39,40]. The need for registration between planning CT and MRI has an inherent and systematic bias, evaluated to 0.5–3.5 mm in prostate and head treatments [41,42]. Although a dosimetric effect of such misalignment is easily conceivable, especially in SRT treatments, its objective quantification remains poorly explored. Dearneley et al. evaluated the risk of a 1% target coverage decrease to a reduction in expected tumor control of 1% for prostate cancer [43]. With the high dose conformity provided by SRT and VMAT, such a dose decrease could theoretically happen often.

With precise delineation and tight PTV margins, delays from the treatment planning to the treatment completion must be as short as possible. A recent brain MRI (<15 days) is essential for the delineation of GTV and it often needs to be renewed to meet this criterion [44]. With our workflow, one patient could be treated within one week of the diagnostic MRI (vs 3 to 4 weeks). However, the thermoplastic mask would need to be molded at the time of the brain MRI.

Regarding the HU intensity comparability, data have already reported using an NN-generated synthetic brain CT scan. Based on a 52-patient cohort, Dinkla et al. reported very small differences between the 2 CTs, with mean absolute errors (MAEs) of 174 +/− 29 HU, 22 +/− 3 HU and 159 +/− 22 for the bone, soft-tissue and air regions, respectively [11]. Similar results were obtained in our cohort using the RMSE. Since the differences are squared before being averaged, the RMSE penalizes larges errors more than MAE and is especially suited for the study of SRT, where a steep dose gradient is expected.

Results regarding the number of treated lesions and the local gamma analysis passing rate must be taken with caution as local gamma analysis might not be relevant for a high number of treated lesions. Furthermore, this sub-set of patients (those with more than three lesions) represents only 6% of the studied population.

Even if our cohort is the largest cohort of MRI-only brain SRT, it lacks prospective validation. Clinical implementation of such pseudo-CT based RT remains rare, and often occurs in clinical trials [17,45,46]. Furthermore, the workflow still relies on a single registration. Despite being performed on clinically validated software, this remains a limitation. Future works should continue to explore the feasibility of pseudo-CT generation without registration, such as Kazemifar’s workflow [12]. Even if an MRI-only brain RT would accelerate SRT planning, the thermoplastic mask would need to be molded when performing the brain MRI. However, once done, a mask can be kept for several months. Finally, we used a 2D-GAN, although 3D-GAN could be of certain interest for synthetic CT generation, especially for such precise radiation techniques.

GTV visualization is critical for some tumors. MRI-linacs have been recently developed and implemented, with significantly better soft-tissue spatial resolution. Adaptative RT is a technique consisting of offline or online dosimetric recalculation at each fraction based on the daily imaging [47,48]. The fast generation of a synthetic CT scan based on daily MRI imaging could allow rapid recalculation with high precision. Extending the results of our study to primary brain tumors (i.e., glioblastoma or benign tumors such as meningioma) is also currently under investigation in our institution.

## 5. Conclusions

Our study is the first to fully compare GAN-generated CT scans from diagnostic brain MRIs and initial CT scans for the planning of brain stereotactic RT. Synthetic CT scans appeared to be equivalent to the planning scan. Their implementation in daily routines could help by reducing treatment delays and misalignments due to iterative registrations. Prospective validation is currently under investigation in our institution to further ensure the place of pseudo-CT scanning for dosimetric considerations.

## Figures and Tables

**Table 1:** Main patients’ characteristics.

Main Patients Characteristics		*N* = 184	%
Gender	Male	105	57.1
	Female	79	42.9
Age median (range)		60 (31–85)	
Primary Histology	Lung	113	61.4
	Breast	30	16.3
	Melanoma	21	11.4
	Kidney	5	2.7
	GI	12	6.5
	Bladder	2	1.1
	Osteosarcoma	1	0.5
Planning target volume (PTV) (cm^3^)	Mean (range)	6.44 (0.37–45.41)	
	Median	3.89	
Number of brain metastases	1	114	62.0
	2–3	59	32.0
	>3	11	6.0
	Mean (range)	1 (1–6)	
Prescription dose	PD1	78	42.4
	PD2	106	57.6

**Table 2:** HU comparability and DRR comparability between the initial CT scan and the synthetic CT scan (root mean square error).

Dose	Bone	Soft-Tissue	DRR (20 Patients)
Mean (HU)	175.50	13.54	86.16
Median (HU)	179.58	13.70	80.30
SD	63.15	1.96	19.80

Abbreviations: HU: Hounsfield unit, DRR: digital reconstructed radiograph.

**Table 3:** Results of the local and global gamma analyses for each set (Overall, Training and Testing) with the 2%/2 mm criteria (10% maximum dose threshold).

Gamma Analysis	Dose	Overall	Training	Testing	*p*
Local Gamma Analysis	Median	99.5	99.6	99.5	0.43
	Mean	99.1	99.3	99.1	
	SD	0.53	0.54	0.54	
Global Gamma Analysis	Median	99.8	99.9	99,8	0.83
	Mean	99.7	99.8	99.7	
	SD	0.39	0.18	2.12	

Abbreviation: SD: standard deviation.

**Table 4:** Dose-volume histogram (DVH) comparisons between the initial dose and the synthetic dose maps for the PTVs.

DVH Feature	Initial			Synthetic			Absolute Difference				Relative Difference
	Median	Mean	SD	Median	Mean	SD	Median	Mean	SD	*p*	%
PTV Volume (mL)	3.89	6.44	7.98								
Dmin (Gy)	22.56	22.20	1.84	22.85	22.46	1.87	0.30	0.26	0.34	0.15	1.17
Dmax (Gy)	32.94	29.89	4.54	33.11	30.28	4.57	0.41	0.39	0.28	0.38	1.30
Dmean (Gy)	28.21	26.83	2.78	28.42	27.19	2.80	0.38	0.37	0.24	0.19	1.38
D2 (Gy)	32.25	29.30	4.34	32.44	29.62	4.64	−0.42	−0.32	1.79	0.47	−1.09
D50 (Gy)	27.99	26.85	2.86	28.19	27.12	3.24	−0.38	−0.27	1.69	0.37	−1.01
D98 (Gy)	24.18	23.89	1.48	24.43	24.09	2.11	−0.32	−0.20	1.51	0.26	−0.84
Reference Isodose Volume (mL)	11.46	14.43	12.94	11.76	15.02	13.34	0.30	0.60	0.72	0.65	4.16
Half Reference Dose Volume (mL)	46.93	61.92	50.05	48.05	62.99	51.34	−1.21	−0.42	1.06	0.83	−0.68
Conformation Index	1.39	1.44	0.22	1.42	1.48	0.25	0.03	0.02	0.06	0.10	4.86
Ihomogeneity1	1.43	1.30	0.19	1.43	1.32	0.19	0	−0.02	0.01	0.28	−1.54
Ihomogeneity2	0.32	0.24	0.15	0.33	0.24	0.15	0.00	−0.01	0.02	1	−4.17
Ihomogeneity3	0.25	0.19	0.11	0.26	0.20	0.17	0.00	−0.01	0.13	0.48	−5.26
Dose Gradient	4.34	4.87	2.03	4.26	4.70	2.00	0.05	0.17	0.51	0.39	3.49

Abbreviations: Dx: dose received by x% of the volume (Gy), SD: standard deviation, Ihomogeneity 1: PTV Dmax/PD (PD: prescribed dose), Ihomogeneity 2: (D2 − D98)/PD, Ihomogeneity 3: (D2 − D98)/D50, Dose Gradient: PD50%/Volume Isodose PD.

## Data Availability

Data available on request due to restrictions (privacy).

## Supplementary Materials

The following are available online at https://www.mdpi.com/2072-6694/13/5/1082/s1. Table S1: Results of the Local and Global Gamma Analyses for each criteria (2%/2 mm, 2%/1 mm and 1%/1 mm), Table S2: DVH comparisons between the Initial dose and the Synthetic dose maps for the Organs at Risks, Figure S1: Example of DRRs comparison: original CT scan (a) and synthetic CT scan (b), Figure S2: Representation of the local Gamma Analysis passing rate (%) depending on the volume of the treated PTV (mL), Figure S3: Representation of the local Gamma Analysis passing rate (%) depending on the number of treated metastases.
