# Supplementary material for: Dosimetric Validation of a GAN-Based Pseudo-CT Generation for MRI-Only Stereotactic Brain Radiotherapy
Source: Cancers (Basel). 2021 Mar 3;13(5):1082. doi: 10.3390/cancers13051082 (PMC7959466; doi:10.3390/cancers13051082)
Supplement: Supplementary file 1 [file cancers-13-01082-s001.pdf]

cancers

MDPI

Supplementary Materials

Dosimetric Validation of a GAN-Based Pseudo-CT Generation for MRI-Only Stereotactic Brain Radiotherapy

Vincent Bourbonne, Vincent Jaouen, Clément Hognon, Nicolas Boussion, François Lucia, Olivier Pradier, Julien Bert, Dimitris Visvikis and Ulrike Schick

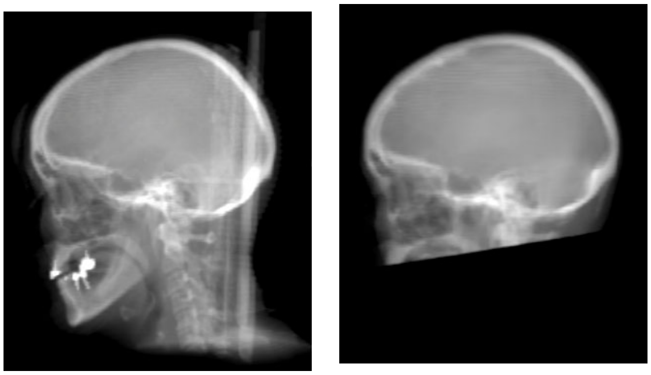

(**a**) (**b**)

Figure S1. Example of DRRs comparison: original CT scan (a) and synthetic CT scan (b).

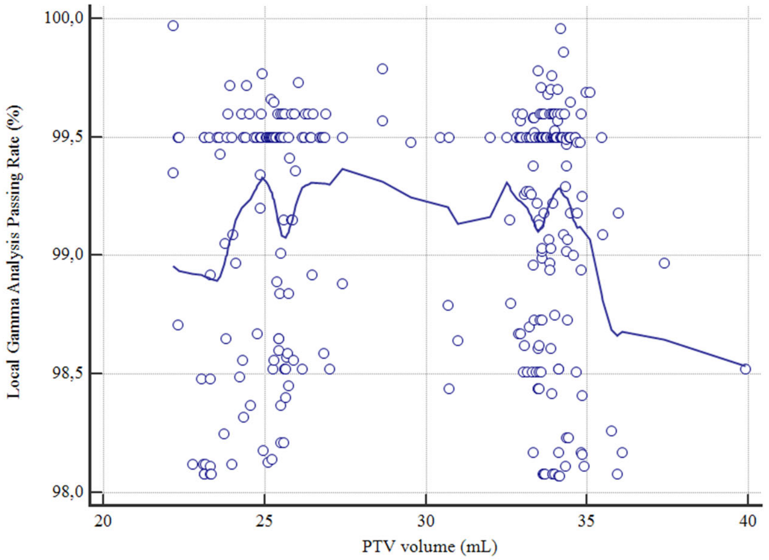

Figure S2. Representation of the local Gamma Analysis passing rate (%) depending on the volume of the treated PTV (mL). Abbreviations: PTV: Planning Target Volume (mL), blue line : Local Regression Smoothing Line with a 20% span.

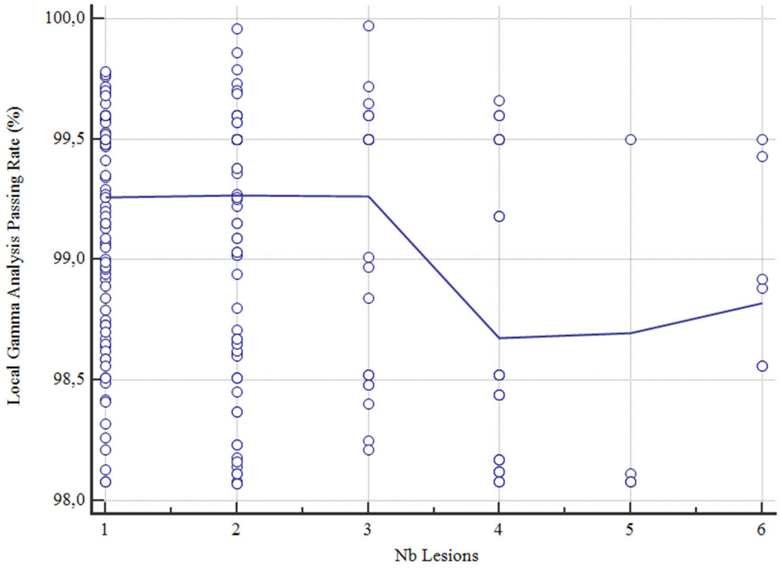

Figure S3. Representation of the local Gamma Analysis passing rate (%) depending on the number of treated metastases. Abbreviations: Nb: number, blue line: Local Regression Smoothing Line with a 20% span

Table S1. Results of the Local and Global Gamma Analyses for each criteria (2% / 2mm, 2% / 1mm and 1% / 1mm).

Gamma Analysis	Dose	2% / 2 mm	2% / 1 mm	1% / 1 mm
Local Gamma Analysis	Median	99.5	98.0	87.1
	Mean	99.1	97.99	87.4
	SD	0.53	0.96	3.66
Global Gamma Analysis	Median	99.8	99.7	99.5
	Mean	99.7	99.6	99.2
	SD	0.39	0.28	5.26

Table S2. DVH comparisons between the Initial dose and the Synthetic dose maps for the Organs at Risks.

Organ at Risk	Dose	**Initial**			**Synthetic**			**Absolute Difference**				**Relative Difference**
		Median	Mean	SD	Median	Mean	SD	Median	Mean	SD	*p*	Mean (%)
**Optic Chiasma**	Dmax (Gy)	1.16	2.59	3.41	1.18	2.63	3.45	0.00	0.04	0.10	*0.90*	1.54
	Dmean (Gy)	0.73	1.74	2.20	0.74	1.77	2.24	0.00	0.03	0.06	*0.89*	1.72
	D2 (Gy)	1.06	2.43	3.15	1.11	2.46	3.19	0.00	−0.03	1.06	*0.89*	−1.23
	D50 (Gy)	0.75	1.71	2.15	0.76	1.73	2.19	0.00	−0.03	0.75	*0.96*	−1.75
	D98 (Gy)	0.51	1.26	1.62	0.52	1.29	1.65	0.00	−0.02	0.51	*0.95*	−1.59
**Left Optic Nerve**	Dmax (Gy)	0.83	1.94	2.31	0.81	1.97	2.36	0.00	0.03	0.09	*0.90*	1.55
	Dmean (Gy)	0.44	1.24	1.54	0.45	1.26	1.56	0.00	0.01	0.04	*0.89*	0.81
	D2 (Gy)	0.79	1.83	2.16	0.77	1.85	2.21	0.00	−0.02	0.79	*0.92*	−1.09
	D50 (Gy)	0.41	1.23	1.54	0.41	1.23	1.56	0.00	0.00	0.41	*1*	0.00
	D98 (Gy)	0.29	0.81	1.13	0.28	0.81	1.14	0.00	0.00	0.29	*0.93*	0.00
**Right Optic Nerve**	Dmax (Gy)	0.82	1.88	2.36	0.84	1.92	2.42	0.00	0.03	0.10	*0.86*	1.60
	Dmean (Gy)	0.39	1.14	1.53	0.40	1.16	1.57	0.00	0.02	0.05	*0.89*	1.75
	D2 (Gy)	0.77	1.77	2.23	0.79	1.79	2.28	0.00	−0.02	0.77	*0.93*	−1.13
	D50 (Gy)	0.39	1.10	1.52	0.38	1.10	1.55	0.00	0.00	0.39	*1*	0.00
	D98 (Gy)	0.25	0.76	1.17	0.25	0.75	1.19	0.00	0.00	0.25	*0.93*	0.00
**Left Eye**	Dmax (Gy)	1.21	1.82	2.05	1.24	1.83	2.09	0.00	0.02	0.09	*0.96*	1.10
	Dmean (Gy)	0.36	0.83	1.05	0.36	0.83	1.05	0.00	0.00	0.02	*1*	0.00
	D2 (Gy)	1.01	1.59	1.78	0.99	1.59	1.79	0.00	0.00	1.01	*1*	0.00
	D50 (Gy)	0.23	0.80	1.10	0.24	0.80	1.09	0.00	0.00	0.23	*1*	0.00
	D98 (Gy)	0.11	0.30	0.52	0.11	0.31	0.53	0.00	−0.01	0.11	*0.85*	−3.33
**Right Eye**	Dmax (Gy)	1.00	1.56	1.87	0.99	1.57	1.88	0.00	0.00	0.05	*0.96*	0.00
	Dmean (Gy)	0.31	0.72	0.97	0.30	0.73	0.98	0.00	0.00	0.02	*0.92*	0.00
	D2 (Gy)	0.78	1.36	1.63	0.78	1.37	1.64	0.00	−0.01	0.78	*0.95*	−0.74
	D50 (Gy)	0.23	0.70	1.02	0.24	0.71	1.03	0.00	0.00	0.23	*0.92*	0.00
	D98 (Gy)	0.10	0.28	0.48	0.10	0.29	0.49	0.00	0.00	0.10	*0.83*	0.00

**Left Lens**	Dmax (Gy)	0.39	0.96	1.27	0.39	0.96	1.26	0.00	0.00	0.04	*1*	0.00
	Dmean (Gy)	0.21	0.74	1.08	0.22	0.74	1.07	0.00	0.00	0.05	*1*	0.00
	D2 (Gy)	0.40	0.97	1.26	0.40	0.98	1.28	0.00	−0.01	0.40	*0.94*	−1.03
	D50 (Gy)	0.25	0.77	1.10	0.26	0.77	1.09	0.00	0.00	0.25	*1*	0.00
	D98 (Gy)	0.15	0.59	0.91	0.15	0.58	0.88	0.00	0.01	0.15	*0.91*	1.69
**Right Lens**	Dmax (Gy)	0.33	0.83	1.11	0.33	0.83	1.11	0.00	0.00	0.04	*1*	0.00
	Dmean (Gy)	0.23	0.61	0.84	0.22	0.62	0.85	0	0.01	0.09	*0.90*	1.64
	D2 (Gy)	0.34	0.83	1.08	0.35	0.83	1.08	0.00	0.00	0.34	*1*	0.00
	D50 (Gy)	0.23	0.63	0.84	0.22	0.63	0.86	0.00	−0.01	0.23	*1*	−1.59
	D98 (Gy)	0.15	0.48	0.67	0.14	0.48	0.68	0.00	−0.01	0.15	*1*	−2.08
**Left Inner ear**	Dmax (Gy)	1.62	3.40	5.93	1.62	3.39	5.60	0.00	0.01	0.71	*0.99*	0.29
	Dmean (Gy)	1.01	2.28	2.59	1.16	2.35	2.64	0.00	0.02	0.05	*0.78*	0.88
	D2 (Gy)	1.51	2.95	3.32	1.45	2.97	3.34	0.00	0.00	0.16	*0.95*	0.00
	D50 (Gy)	1.11	2.37	2.67	1.04	2.40	2.69	0.00	−0.01	0.11	*0.91*	−0.42
	D98 (Gy)	0.85	1.89	2.18	0.77	1.91	2.22	0.00	−0.01	0.09	*0.95*	−0.53
**Right Inner Ear**	Dmax (Gy)	1.26	2.41	3.50	1.30	2.48	3.57	0.00	0.02	0.10	*0.84*	0.83
	Dmean (Gy)	0.73	1.83	2.71	0.77	1.88	2.75	0.00	0.02	0.04	*0.85*	1.09
	D2 (Gy)	1.26	2.47	3.42	1.33	2.51	3.46	0.00	−0.01	0.09	*0.91*	−0.40
	D50 (Gy)	0.93	1.94	2.75	0.87	1.97	2.78	0.00	−0.01	0.07	*0.91*	−0.52
	D98 (Gy)	0.62	1.55	2.22	0.58	1.58	2.26	0.00	−0.01	0.06	*0.89*	−0.65
**Hypophysis**	Dmax (Gy)	1.38	2.82	3.14	1.40	2.87	3.18	0.00	0.01	0.06	*0.87*	0.35
	Dmean (Gy)	0.78	2.10	2.32	0.77	2.13	2.34	0.00	0.01	0.04	*0.90*	0.48
	D2 (Gy)	1.37	2.69	2.87	1.41	2.72	2.90	0.00	−0.02	0.12	*0.92*	−0.74
	D50 (Gy)	0.83	2.11	2.29	0.91	2.13	2.31	0.00	−0.01	0.08	*0.93*	−0.47
	D98 (Gy)	0.59	1.58	1.78	0.67	1.61	1.80	0.00	−0.01	0.07	*0.86*	−0.63
**Brainstem**	Dmax (Gy)	5.46	7.83	7.71	5.74	7.90	7.77	0.02	0.08	0.19	*0.93*	1.02
	Dmean (Gy)	1.38	2.46	2.90	1.39	2.48	2.92	0.00	0.02	0.05	*0.94*	0.81
	D2 (Gy)	0.00	1.71	5.79	0.00	1.74	5.81	0.00	0.02	0.16	*0.96*	1.17
	D50 (Gy)	4.04	6.41	6.91	4.10	6.45	6.98	−0.01	−0.04	0.18	*0.95*	−0.62
	D98 (Gy)	0.89	2.19	2.89	0.89	2.21	2.91	0.00	−0.02	0.05	*0.94*	−0.91
	Dmax (Gy)	0.11	0.23	0.34	0.10	0.23	0.34	0.00	0.00	0.03	*1*	0.00
**Brain**	Dmin (Gy)	0.01	0.03	0.05	0.01	0.03	0.05	0.00	0.00	0.01	*1*	0.00
	Dmax (Gy)	33.39	30.43	4.57	33.75	30.85	4.60	0.44	0.42	0.26	*0.35*	1.38

	Dmean (Gy)	2.11	2.40	1.36	2.13	2.43	1.38	0.03	0.03	0.03	*0.82*	1.25
	V10 (mL)	3.72	4.97	4.27	3.80	5.10	4.34	0.09	0.12	0.11	*0.76*	2.41
	V21 (mL)	1.02	1.24	1.06	1.06	1.28	1.09	0.03	0.04	0.05	*0.70*	3.23
	D2 (Gy)	14.39	14.87	5.45	14.60	15.07	5.58	−0.21	−0.20	0.42	*0.71*	−1.34
	D50 (Gy)	0.43	1.01	1.24	0.44	1.02	1.25	0.00	−0.01	0.04	*0.93*	−0.99
	D98 (Gy)	0.04	0.07	0.09	0.03	0.07	0.09	0.00	0.00	0.03	*1*	0.00

Abbreviations: VxGy : volume receiving x Gy (mL), Dmax : maximum dose, Dmean : mean dose, Dmin : minimum dose, Dx :dose received by x % of the volume (Gy), SD : standard deviation.
